# Development of postural adjustments during reaching in typically developing infants from 4 to 18 months

**DOI:** 10.1007/s00221-012-3121-9

**Published:** 2012-05-24

**Authors:** Lieke C. van Balen, Linze Jaap Dijkstra, Mijna Hadders-Algra

**Affiliations:** Department of Paediatrics, Developmental Neurology, University Medical Center Groningen, University of Groningen, Hanzeplein 1, 9713 GZ Groningen, The Netherlands

**Keywords:** Postural control, EMG, Reaching, Infants

## Abstract

Knowledge on the development of postural adjustments during infancy, in particular on the development of postural muscle coordination, is limited. This study aimed at the evaluation of the development of postural control during reaching in a supported sitting condition. Eleven typically developing infants participated in the study and were assessed at the ages of 4, 6, 10 and 18 months. We elicited reaching movements by presenting small toys at an arm’s length distance, whilst activity of multiple arm, neck and trunk muscles was recorded using surface EMG. A model-based computer algorithm was used to detect the onset of phasic muscle activity. The results indicated that postural muscle activity during reaching whilst sitting supported is highly variable. Direction-specific postural activity was inconsistently present from early age onwards and increased between 10 and 18 months without reaching a 100 % consistency. The dominant pattern of activation at all ages was the ‘complete pattern’, in which all direction-specific muscles were recruited. At 4 months, a slight preference for top-down recruitment existed, which was gradually replaced by a preference for bottom-up recruitment. We conclude that postural control during the ecological task of reaching during supported sitting between 4 and 18 months of age is primarily characterized by variation. Already from 4 months onwards, infants are—within the variation—sometimes able to select muscle recruitment strategies that are optimal to the task at hand.

## Introduction

In the first 18 months of life, an infant’s motor skills develop at an astounding rate: the child learns to balance the head, to reach and grasp, to sit, to crawl and to walk. It is therefore not surprising that postural control improves rapidly during this period. However, our knowledge on the development of postural control during infancy, in particular on the development of postural muscle coordination, is limited.

In general, knowledge on neurobiological substrate and mechanisms underlying motor development is scarce. As a result, various theoretical models are used to explain motor development. A well-known theoretical framework is the Dynamic Systems Theory (DST; Thelen and Smith 1994; Adolph and Robinson 2008). This theory considers motor development as a dynamic system in which motor behaviour emerges as a result of a complex interaction between the intrinsic properties of the body, the results of previous experiences and environmental factors. The Neuronal Group Selection Theory (NGST, Hadders-Algra 2010) is an alternative theoretical framework. DST and NGST partially overlap. The two theories share the opinion that motor development is a non-linear process with phases of transition, a process that is affected by many factors. Both theories acknowledge the importance of experience and the relevance of context. But the two theories differ in their opinion on the role of genetically determined neurodevelopmental processes. Genetic factors only play a limited role in DST, whereas in NGST genetic endowment, epigenetic cascades and experience play equally prominent roles (Hadders-Algra 2010). According to NGST, typical motor development is characterized by two phases of variability. Development starts with primary variability: the nervous system explores its repertoires of possibilities. The borders of the repertoires are determined by genetic instructions. Exploration of the repertoires results in abundant variation in motor behaviour and in a wealth of self-produced afferent information. During the phase of primary variability, afferent information is not used for the adaptation of motor behaviour to the specifics of the situation. This alters during the phase of secondary variability. During secondary variability, afferent information associated with exploration and trial-and-error experiences is used for the selection of the optimal movement strategy for each situation (Hadders-Algra 2010). According to NGST, the consequences of an early lesion of the brain are twofold: (a) a reduction in the size of the repertoire (reduced variation) and (b) a limited ability to select the best strategy from the repertoire (reduced variability; Hadders-Algra 2010). Here, it is important to note that the word ‘variability’ has been used in different ways in scientific literature. For instance, ‘variability’ has been used to denote variation; following this word use, reduced variability has been considered a sign of dysfunction. Variability has also been used to denote the ability to vary; following this word use, an excess of variation in motor output (also termed excessive variability) has been considered non-optimal behaviour (Harbourne and Stergiou 2009; Dusing and Harbourne 2010). In this study, we follow Hadders-Algra (2010) where variation refers to the size of the motor repertoire and variability to the ability to vary (i.e. the ability to select different strategies from the motor repertoire in order to adapt motor output to the specifics of the situation).

This article aims to study the development of postural control in infancy. Both in adults and children, control of posture has mainly been studied during standing and walking. Knowledge on the early development of postural control, such as during the development of sitting, is limited. Harbourne and Stergiou (2003) studied postural control in infants just before and after they developed the ability to sit independently and found that as infants learn to sit, the approximate entropy (a measure of variation) of the sway path of their centre of pressure decreased, indicating that the infants had learned to select those strategies that were optimal for sitting independently. However, centre of pressure data do not furnish information on the strategies used by the nervous system to achieve the various sway paths. Data on muscle recruitment may provide such insight.

### Muscle recruitment strategies during postural development

Successful control of body posture is accomplished by activating the proper muscles at the proper time with an optimal contraction strength. Earlier studies into postural muscle activation strategies have revealed several parameters with which the development of postural control can be described.

#### Direction specificity

According to Forssberg and Hirschfeld (1994), direction specificity is the first or basic level of postural control. Direction specificity means that when equilibrium is threatened by a forward sway of the body, the muscles on the dorsal side of the body are primarily activated in order to maintain balance, and when equilibrium is threatened by a backward sway, the muscles on the ventral side are primarily activated. The ability to recruit directionally appropriate muscles has been shown to exist already in early infancy: from the age of one month onwards, infants consistently use direction-specific postural adjustments in response to external perturbations of balance (Forssberg and Hirschfeld 1994; Hedberg et al. 2004, 2005; Washington et al. 2004). In terms of NGST, this suggests that infants are endowed with a direction-specific repertoire of postural adjustments, that is, with variation in direction-specific adjustments. However, research findings are less clear in case of internally triggered movements, such as reaching. Van der Fits and Hadders-Algra (1998) and Van der Fits et al. (1999a, b), who longitudinally assessed postural adjustments during reaching between 3 and 18 months of age, reported that postural adjustments are direction-specific from the moment that successful reaching movements emerge, which happens around 4–5 months. A more recent study indicated, however, that only approximately half of the reaching movements at 4 and 6 months are accompanied by direction-specific postural adjustments (De Graaf-Peters et al. 2007). The difference between the older and more recent data is explained by the use of a more stringent definition of direction specificity in the latter: in the older studies of Van der Fits et al., a trial was classified as direction-specific when direction specificity was present at one of the body levels (e.g. neck, trunk or legs) recorded irrespective of the organization of postural activity at other levels of the body. In the recent study of De Graaf-Peters et al., trials were only considered direction-specific when postural activity at all levels of the body was direction-specific (cf., Hedberg et al. 2004, 2005). Using the same stringent definition, Van der Heide et al. (2003) found that from the age of 2 years onwards children consistently use direction-specific postural adjustments whilst reaching. The data may imply that at early age the direction-specific networks are present, but not recruited consistently during the early phases of the development of reaching. A question that remains is at which age between 4 months and 2 years reaching movements are consistently accompanied by direction specificity.

Forssberg and Hirschfeld (1994) described that the second level of postural control consists of the ability to adapt direction-specific adjustments to the specifics of the situation. In terms of NGST, the parameters of the second level of control are parameters of variability. Some of the parameters that can be distinguished are:

#### Complete pattern

One way to adapt postural adjustments is the selection of particular direction-specific muscles or a particular combination of direction-specific muscles. The study of De Graaf-Peters et al. (2007) indicated that at 4 months the number of direction-specific muscles that are recruited is highly variable. Already at 6 months, some selection occurs: the ‘complete pattern’, that is, the pattern in which all recorded direction-specific muscles are recruited, becomes a more prominent pattern. It also has been shown that the complete pattern is the dominant pattern whilst reaching in a sitting position during the second postnatal year (Van der Heide et al. 2003; Hadders-Algra 2008). This preference for the complete pattern in early infancy may be related to the difficulty of the balance problem that is encountered, as this preference disappears after the second year, when infants have mastered walking (Hadders-Algra 2008).

#### Recruitment order

Our knowledge on the development of the recruitment order of direction-specific muscles during infancy is limited. Variation appears to be the major characteristic of recruitment order at early age. Varied recruitment is evident at 4 months of age. At 6 months, infants who sit supported show a mild preference for top-down recruitment (De Graaf-Peters et al. 2007). However, independently sitting 8- to 10-month-old infants demonstrate a slight preference for bottom-up recruitment (Hadders-Algra et al. 1996a, b; Van der Fits et al. 1999b). Longitudinal data on the development of the recruitment order of direction-specific muscles in supported sitting are lacking. Recruitment order of postural muscles in independently sitting children beyond the age of 10 months is mainly characterized by variation (Van der Heide et al. 2003).

#### Anticipatory activation

In EMG-studies of postural control during reaching, anticipatory postural activity has been defined as the occurrence of activity in postural muscles prior to the activation of the first arm muscle that initiates the reaching movement (the ‘prime mover’). Anticipatory muscle activation is a form of postural fine-tuning that heavily relies on feed-forward processing (Massion 1992). The studies of Van der Fits and Hadders-Algra (1998), Van der Fits et al. (1999a, b) indicated that, from 15 months onwards, infants show a significant increase in the use of anticipatory postural activity during reaching whilst sitting.

One of the challenges of studying postural control in an ecological setting is that the task-related activation in the EMG signal is more or less ‘hidden’ in the noise of other movements. Previous studies using the ecological design of reaching movements to study postural control (Van der Fits et al. 1999a, b; De Graaf-Peters et al. 2007) used a separate video analysis to indicate the approximate time of the start of the reaching movement in the EMG signal, combined with a fixed detection level threshold (compared with a long-term mean baseline activity) to identify task-related activation. The latter implied that spontaneous activity prior to the reaching movement had a relatively large impact on whether or not reaching-related postural EMG-activity could be detected in the window of analysis. In order to improve the accuracy of data analysis, we developed a software program (PedEMG). PedEMG has two advantages. Firstly, it integrates video and EMG analysis. The simultaneous view of infant behaviour and EMG signals allows for a better selection of trials that are not affected by simultaneously occurring additional activities. Secondly, the program does not use a fixed-threshold algorithm for onset detection, but a dynamic threshold statistical algorithm as the latter performs better in noisy signals than the former (Staude and Wolf 1999).

The aim of this study is to increase our understanding of postural development in the ecological situation of reaching during supported sitting between 4 and 18 months. To this end, we carried out a longitudinal study with eleven typically developing infants who were assessed at 4, 6, 10 and 18 months in which the novel program PedEMG was applied. We opted to study the infants at specific ages instead of at specific functional levels. Either option has advantages and disadvantages, which are related to the fact that development is the net result of the continuous interaction between genetic endowment and external influences, including experience. We chose the age approach as previous studies indicated that the relationships between functional achievements such as ‘being able to sit without help’ and parameters of postural control are only weakly associated (Hadders-Algra et al. 1996a; Van der Fits et al. 1999b). In addition, we aim to continue our research with infants at high risk of developmental disorders. In general, clinical follow-up of high-risk infants involved assessment at specific ages and not at specific abilities. We did, however, include the development of motor milestones in our assessments in order to explore possible associations between functional motor development and the development of postural control.

We addressed the following questions: (1) At what age do infants consistently show direction-specific postural adjustments when reaching? (2) Can we replicate the finding of an increased selection of the complete pattern with increasing age similar to that occurring during external perturbations in a sitting position (Hedberg et al. 2005)? (3) Does recruitment order of the direction-specific muscles during reaching whilst sitting supported change with increasing age, that is, do infants develop a preference for top-down (cf. De Graaf-Peters et al. 2007) or bottom-up recruitment (Van der Fits et al. 1999b; Van der Heide et al. 2003), or is recruitment primarily characterized by variation? (4) Do infants between 4 and 18 months of age exhibit anticipatory postural activity when reaching? (5) Are the postural control parameters mentioned above (i.e. direction specificity, presence of the complete pattern, recruitment order and anticipatory activation) associated with the achievement of milestones in sitting and grasping behaviour?

## Methods

### Subjects

Eleven full-term healthy infants (six boys and five girls) were recruited from amongst acquaintances of the investigators and participated in the study. They were assessed longitudinally at the ages of 4, 6, 10 and 18 months. One infant was first included at the age of 6 months and thus did not participate at 4 months; data of another infant at 10 months are missing because of an inappropriate behavioural state. The infants’ gestational age at birth varied from 37 weeks and 4 days to 42 weeks postmenstrual age (median value: 40.5 weeks); birth weight from 3,000 to 4,000 g (mean 3,597 g; SD 322 g). All children had a typical motor development. The parents of the infants gave informed consent and the procedures were approved by the ethics committee of the University Medical Center Groningen.

### Protocol

The infants were tested in a supported sitting position. The infants sat either in an infant chair with back support and a horizontal bar at the level of the upper abdomen that provided additional support at the front, or on their parents’ lap, with their legs in a semi-flexed position. The latter position was only applied at 18 months. It was used to obtain the infant’s cooperation. Care was taken that the sitting position on the parents lap closely resembled that in the infant chair; in both situations the infants used little back support.

Reaching was elicited by presenting small, attractive toys in the midline at an arm’s length distance. Toys were only presented when the infant was in a calm and alert behavioural state. For each position, we aimed at recording at least ten reaching movements with the right arm, but when the infant became fussy or tired the session was shortened. The reaching session took about 30 min. After each session, the child’s neurological integrity was confirmed using the age-specific Touwen Infant Neurological Examination. With this examination, minor neurological dysfunction can be detected reliably with an inter-assessor agreement of κ = 0.83 (Hadders-Algra et al. 2010). The assessor was aware of the fact that she assessed a typically developing infant, but she was blinded to the EMG-data. The assessment also included evaluation of milestones. In this study, we used the development of sitting (trichotomized as unable to sit independently, able to sit independently for a few seconds, able to sit independently for ≥10 s) and grasping (scored as either palmar grasp, radial palmar grasp, scissor grasp, inferior pincer grasp or pincer grasp).

### EMG and kinematic recordings

EMG was measured continuously during the testing session with bipolar surface electrodes with an inter-electrode distance of 14 mm on the bellies of the following muscles on the right side of the body: deltoid (DE), pectoralis major (PM), biceps brachii (BB), triceps brachii (TB), neck flexor (NF, sternocleidomastoid), neck extensor (NE), rectus abdominis (RA), thoracal extensor (TE), lumbar extensor (LE), rectus femoris (RF) and hamstrings (HAM). DE, PM, BB and TB are referred to as arm muscles, NF, NE, RA, TE, LE, RF and HAM as postural muscles. Leg muscle activity was not analysed in this study, as previous studies (Van der Fits et al. 1999a, b; Van der Heide et al. 2003) indicated that in typically developing sitting infants leg muscle activity is not related to postural control. Leg muscle activity was recorded to obtain reference values for studies on the development of postural control in atypically developing infants, in whom leg muscle activity during reaching whilst sitting may be related to postural control.

Approximately halfway through the experimental project, the laboratory environment was updated, so that six of the sessions at the age of 10 months and all of the sessions at the age of 18 months were recorded using updated recording equipment and software. For all sessions, EMG signals were acquired by means of an electro-physiological front-end amplifier (Twente Medical Systems International, Enschede, The Netherlands). The EMG signal was recorded at a sampling rate of 500 Hz. Before the update of the laboratory environment, EMG pre-processing and recording was performed with the software program POLY (Inspector Research Systems, Amsterdam, The Netherlands). After the update, the software program Portilab (Twente Medical Systems International, Enschede, The Netherlands) was used for this purpose. To ensure identical data analysis in both cases, raw data were exported from both programs and analysed with the same software program (PedEMG, see “Video and EMG analysis”). The sessions were recorded on video, before the system update as a split-screen recording from a lateral and frontal view of the infant, after the update in a three-camera set-up from a frontolateral, lateral and posterolateral view. The video registrations were time-coupled to the EMG recordings, both before and after the laboratory update.

### Video and EMG analysis

The video was used to select movements in an appropriate attentional state, performed with the right arm or with both arms which occurred in response to toy presentation. It was also used to evaluate sitting position and postural support when the infant was tested on the parent’s lap: trials were excluded when the position of the child and/or the amount of postural support given by the parent was notably different from the intended position in the infant chair. Movements were classified as pre-reaching movements (Trevarthen 1984), reaching movements which did not end in toy contact, reaching movements which did end in toy contact and reaching movements which ended in grasping of the toy.

EMG analyses were carried out using the PedEMG program (Developmental Neurology, University Medical Center Groningen, The Netherlands). This program uses the model-based computer algorithm of Staude and Wolf (1999) to detect significant bursts of phasic EMG-activity. As this algorithm is especially suitable for detecting onsets in signals with both high and low signal-to-noise ratios (Staude and Wolf 1999), using this algorithm ensured detection of the onset at all ages studied. In short, the algorithm uses a dynamic process model for the EMG signal and defines the onset of a burst of phasic activity as an abrupt change in the model’s parameters. The detection procedure is a statistical decision element that uses a log-likelihood-ratio test to detect the time at which this parameter change occurred. Staude and Wolf (1999) have shown that the algorithm outperforms traditional methods of event detection in statistical simulations.

When appropriate, we first corrected the signals for interference from artefacts and heart muscle activity of the infant, before applying the detection algorithm. Artefact correction consisted of removal of signal peaks that had amplitudes of more than 100 times the amplitude of the average absolute signal. Signals with interference from 50 Hz noise sources were filtered with a fifth-order band stop filter (Chebyshev) at 50 Hz and its higher harmonics. Heart muscle activity, which is present in EMGs of muscles in the relative vicinity of the heart, was identified by using pattern recognition algorithms searching for the regularly repeating pattern and specific shape of QRS-complexes.

After pre-processing, we applied the onset detection algorithm to the intervals containing the selected reaching movements, starting 3 s before and ending 8 s after the start of the reaching movement on the video. The parameters for the detection algorithm were adjusted manually for each muscle signal in order to correct for variations in signal characteristics such as noise levels and overall signal amplitude. Identification of arm muscle activity related to the reaching movement was accomplished by simultaneously playing the video and EMG recordings and marking the current video frame in the EMG signal. Onset detection in the arm muscles started at the onset of the reaching movement in the video and ended when the reaching movement on the video ended. The activity of the neck, trunk and leg muscles was considered to be related to the arm movement when increased muscle activity was found within a time window of 100 ms before activation of the prime mover (the arm muscle that was activated first) and the end of the reaching movement. If the duration of the reaching movement was longer than 1,000 ms, the interval was truncated at 1,000 ms.

For each infant at each age, the following parameters were calculated: (1) percentage of direction-specific trials at neck and/or trunk level; direction specificity meant that the ‘direction-specific’ (i.e. dorsal) muscle was recruited prior to the ventral muscle or without activation of the antagonistic ventral muscle. The other EMG-parameters were calculated only for trials with direction-specific postural activity at trunk level: (2) patterns of postural adjustments, where patterns consist of the specific combinations in which direction-specific muscles are activated in concert. Specific attention was paid to the occurrence of the ‘complete pattern’, that is, the pattern in which all recorded direction-specific muscles are recruited; (3) the preference pattern defined as the pattern that was used most frequently; (4) the latencies of recruitment of postural muscles, defined as the time interval between the onset of the prime mover and the onset of activity in the postural muscle. For each infant at each age, median latency values were calculated; (5) the percentage of trials with a top-down, bottom-up, simultaneous and mixed order of recruitment. Recruitment order could only be determined when at least two direction-specific muscles showed significant phasic activity. If two muscles were activated within an interval of 20 ms, recruitment was considered to be simultaneous; (6) Percentage of trials with anticipatory postural activity at the neck and/or trunk level (i.e. activation starting within 100 ms before the start of the prime mover).

### Statistics

Statistical analyses were performed using the computer package SPSS (version 16.0). Due to the non-normal distribution of the data—a finding, which is typical for infancy—non-parametric statistics were used. When possible, we used the Wilcoxon matched pairs test for the analyses. For comparisons where, due to loss of data, this was not possible, the Mann–Whitney *U* test and the Kruskal–Wallis test were used. Chi-square test for trend was used to evaluate the association between age and the presence of specific preference patterns. Throughout the analyses, differences with a *p* < 0.05 were considered statistically significant.

## Results

Preliminary data analysis indicated that EMG-activity of the 18-month-old infants who were assessed in the infant chair did not differ from that of their peers who had been assessed on their parent’s lap. For instance, the median values of direction specificity were 90 % in the infant chair and 86 % in the lap sitting positions. Therefore, the data of these infants were pooled. At the age of 4 months, 3 infants produced pre-reaching movements in some (1 infant) or all (2 infants) of the trials. Testing for differences in direction specificity at trunk level revealed that the pre-reaches were less often accompanied by direction-specific postural activation than actual reaching movements (median values 33 and 63 %). Notwithstanding the fact that this difference failed to reach significance (due to the small number of pre-reaches (*p* = 0.134)), we decided to exclude these trials from the analysis. The preliminary analysis did indicate that postural activity during reaches with or without toy contact or with or without grasping was similar. Therefore, these trials were pooled for further analysis. Table 1 shows the number of trials included in the analyses.

**Table 1:** Number of infants and number of trials assessed in the two different positions at each age

Position	4 months		6 months		10 months		18 months	
	Infants	Trials^a^	Infants	Trials^a^	Infants	Trials^a^	Infants	Trials^a^
Chair	8	9.5 (5–16)	11	11 (7–30)	7	15 (8–17)	2	17 (5–29)
Lap	0	0	0	0	0	0	9	17 (10–24)

^a^Median (range)

**Fig. 1:**
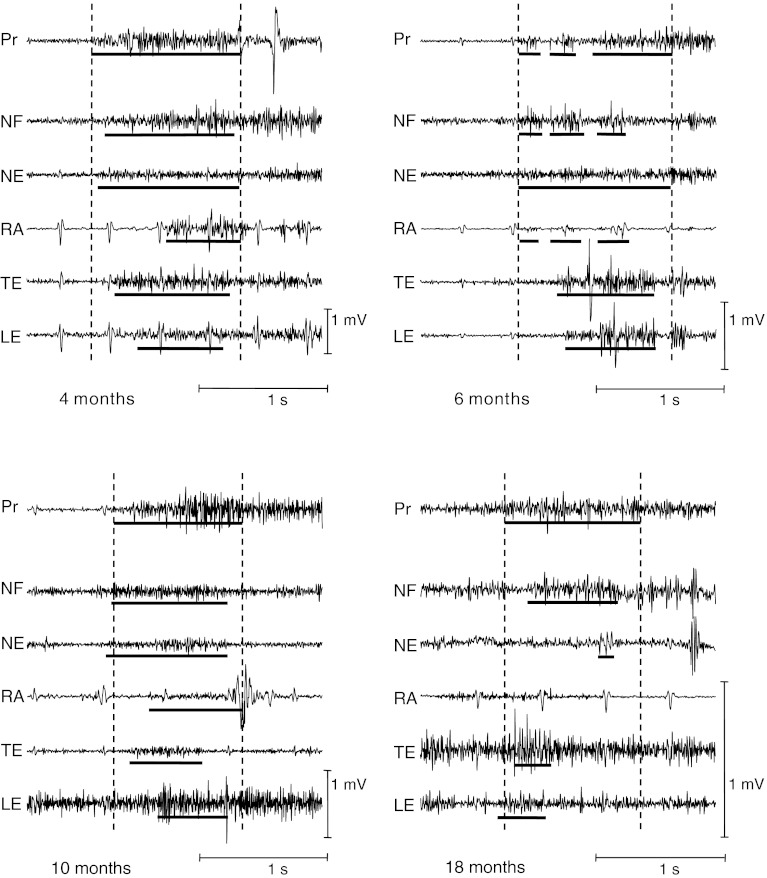
Examples of EMG recordings showing postural adjustments during reaching movements. *Pr* prime mover (the arm muscle initiating the reaching movement), *NF* neck flexor, *NE* neck extensor, *RA* rectus abdominis, *TE* thoracal extensor, *LE* lumbar extensor. *Upper left panel:* a trial at 4 months. Postural adjustments are direction-specific both at neck and trunk level; the direction-specific muscles were recruited in a top-down order. *Upper right panel:* trial at 6 months. Activation in the trunk is not direction-specific; the neck flexor and neck extensor are activated simultaneously. *Lower left panel:* trial at 10 months. This trial shows direction-specific anticipatory activation at neck level but not direction-specific activation at trunk level. *Lower right panel:* trial at 18 months. Activation in the trunk is direction-specific, activation in the neck is not. Recruitment order of the dorsal postural muscles is bottom-up. The lumbar extensor shows anticipatory activation

At all testing ages postural activity accompanying reaching movements was characterized by variation, that is, variation in which muscles were activated, at what point in time and how much. Examples are provided in Fig. 1.

### Direction-specific postural control

**Fig. 2:**
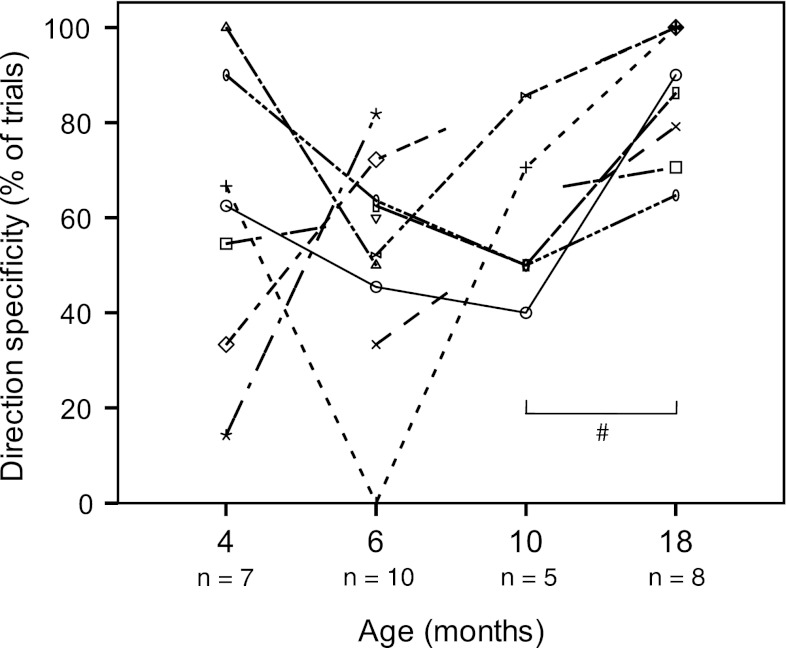
Percentage of direction-specific trials at trunk level during infancy. *Lines* denote individual developmental trajectories; *each line* represents one infant. # Wilcoxon matched pairs test, *p* = 0.031

**Fig. 3:**
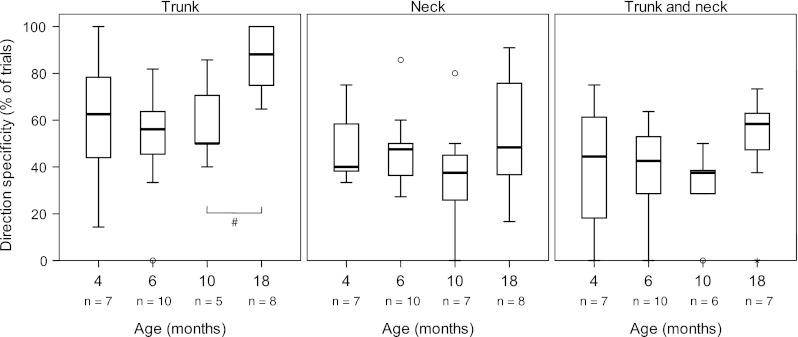
Percentage of direction-specific trials at various ages. *Left panel:* direction specificity in the trunk muscles. *Middle panel:* direction specificity in the neck muscles. *Right panel:* direction specificity present in both neck and trunk. # Wilcoxon matched pairs test, *p* = 0.031. Low numbers are due to missing values in trials where the quality of one or more EMG signals was too poor to allow analysis

Between 4 and 10 months of age, the percentage of reaches accompanied by direction-specific postural activity at trunk level was 50–63 % (median values), with a large variation between and within infants. At 18 months, however, there was a marked increase in direction specificity in the trunk to 88 % (median value, *p* = 0.031; Figs. 2, 3). Direction specificity at neck level remained at 40–50 % of trials throughout infancy. The percentage of trials with direction-specific activation in both neck and trunk was 38–44 % (median values) between 4 and 10 months. It rose to 58 % at 18 months, but the increase did not reach statistical significance (*p* = 0.075; Fig. 3).

### Use of the complete pattern

**Fig. 4:**
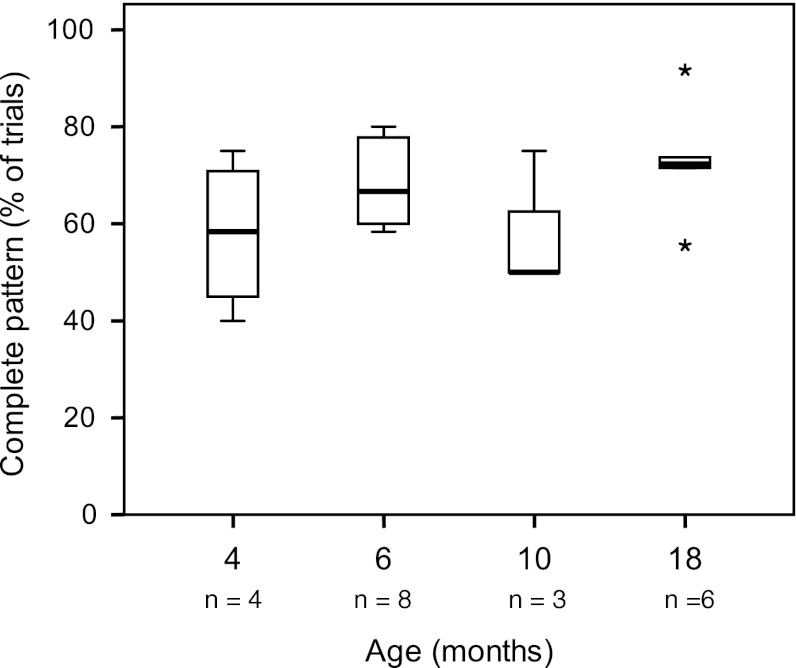
Proportion of trials with the complete pattern from 4 to 18 months. Throughout the age period studied, the complete pattern was the dominant pattern of recruitment of virtually all infants. Some numbers were low as analysis was restricted to direction-specific trials; in addition see legends Fig. 3

At all ages, the majority of infants had a preference for the use of the complete pattern (Fig. 4). This pattern was dominant to such a degree that all infants at all ages, except one 4-month-old infant, used this pattern in at least 50 % of the trials. This preference did not change with increasing age.

### Latency to recruitment of postural muscles

At all ages, the latencies to recruitment of the direction-specific dorsal muscles were largely variable (Table 2). The median values of the latencies of neck flexor and neck extensor muscles varied from 84 to 182 ms, those of the trunk extensors from 43 to 178 ms, which was substantially shorter than those of the rectus abdominis muscle (256–399 ms). The values reflect that the analysis of the latencies were restricted to trials with direction-specific activity. The latencies did not change with increasing age.

**Table 2:** Latencies of direction-specific postural activation

Age	Neck flexor	Neck extensor	Rectus abdominis	Thoracal extensor	Lumbar extensor
4 months	120 [−68, 318]	143 [20, 448]	399 [270, 558]	74 [−30, 256]	178 [−13, 384]
6 months	122 [15, 511]	84 [−48, 438]	384 [174, 596]	114 [77, 162]	84 [−1, 377]
10 months	117 [73, 190]	182 [−19, 349]	256 [125, 473]	43 [6, 259]	54 [28, 460]
18 months	157 [−14, 278]	123 [16, 228]	388 [312, 562]	103 [69, 216]	102 [−68, 415]

Results are displayed as median [range], in milliseconds

### Recruitment order

**Fig. 5:**
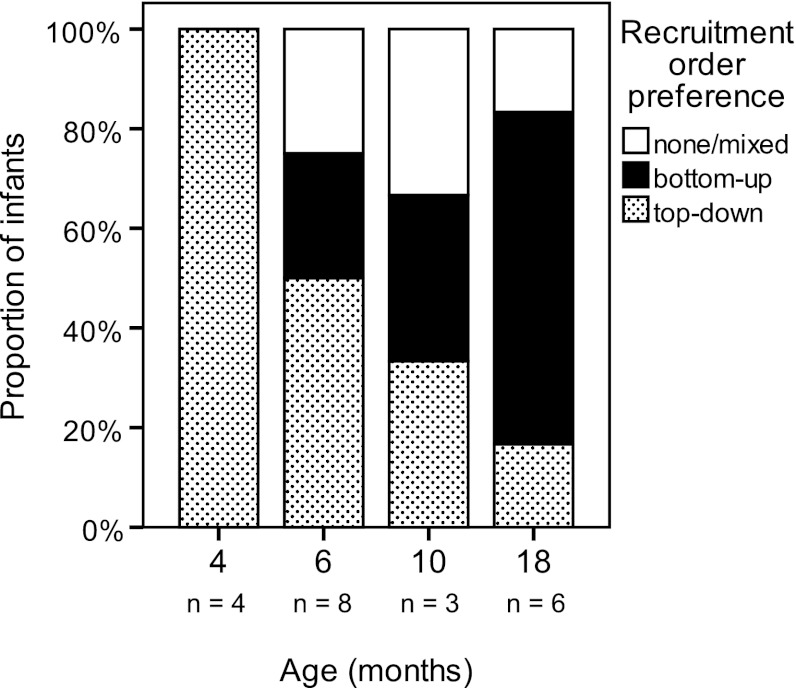
Preferences for bottom-up and top-down recruitment during infancy. The preference pattern of an infant is the most frequently used pattern of that infant. Chi-square for trend: top-down *p* = 0.029, bottom-up *p* = 0.034. Some numbers were low as analysis was restricted to direction-specific trials; in addition see legends Fig. 3

Also recruitment order of the direction-specific postural muscles was characterized by variation: the majority of infants used a mixture of top-down, bottom-up and mixed recruitment of the postural muscles throughout infancy. However, within the variation, the following developmental trend was observed in the infant’s most frequently used recruitment pattern. With increasing age, the bottom-up pattern became increasingly often the infant’s most frequently used recruitment order, at the cost of the initially preferred top-down recruitment (Fig. 5, Chi-square for trend: bottom-up *p* = 0.034, top-down *p* = 0.029).

### Anticipatory postural control

**Fig. 6:**
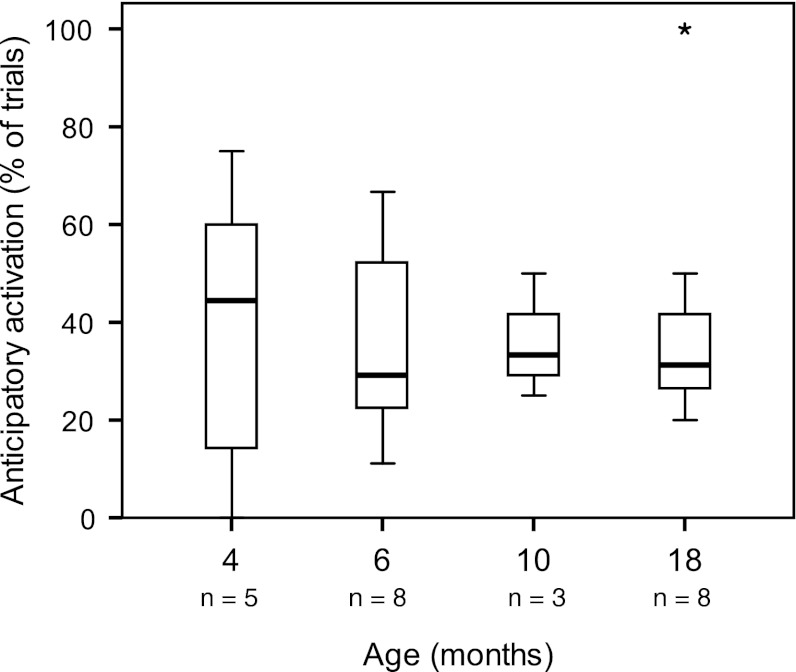
Percentage of trials with anticipatory activation at trunk level during infancy. Some numbers were low as analysis was restricted to direction-specific trials; in addition see legends Fig. 3

Anticipatory activation was found in approximately one-third of the trials (median values) and did not change with age (Fig. 6).

### Association between postural parameters and clinical milestones

At the age of 4 months, only two infants could sit without support for a few seconds. The postural control data of these two ‘almost-sitters’ did not seem to differ from those of their non-sitting peers. At 6 months, two infants could not sit independently, four infants were able to sit independently for a few seconds, and five infants could sit independently for more than 10 s. The infants who were able to sit independently for more than 10 s (*n* = 4) tended to exhibit more top-down recruitment (45 %) than those who were not (*n* = 4; 22 %, median values), but this result did not reach statistical significance (*p* = 0.114). The other postural parameters did not vary with the ability to sit independently. At 10 and 18 months, all infants could sit independently.

The exploration between the relationship between the development of grasping and postural control also only showed interesting trends at the age of 6 months. The infants who had developed the scissor grasp (*n* = 2) tended to show more often top-down recruitment than those how had not developed the scissor grasp (*n* = 6) (58 vs. 37 %, Mann–Whitney *p* = 0.071).

## Discussion

Our results illustrate that postural adjustments during reaching in infancy are characterized by variation and an inconsistent use of direction specificity. Within the variation, two developmental changes emerged. First, the degree of direction specificity during reaching in a supported sitting condition increased between the age of 10 and 18 months. Second, recruitment order gradually changed from a preference for top-down to a preference for bottom-up, reflecting the infant’s ability to adapt in an age-specific way to the postural challenges associated with reaching (variability).

The strength of our study is the application of a longitudinal approach in a natural setting (i.e. during reaching whilst sitting). Moreover, we improved the accuracy of data analysis by integrating video and EMG analysis and using a dynamic statistical algorithm for onset detection in the EMG signals. However, our study has four limitations. First, the number of infants with sufficient data for all parameters at the ages of 4 and 10 months was relatively small. This was caused mainly by (1) the selection of trials of reaches with direction-specific postural activity for the assessment of the fine-tuning of postural control (muscle activation patterns and recruitment order), and (2) in particular at 10 months—the difficulty of keeping all surface electrodes properly attached during the entire session: infants at this age are aware of the strange situation of the recording and have the ability to explore the presence of electrodes and cables, but they do not have the cognitive ability to understand the information that it is better to leave the recording devices in place. This caused different sample sizes for different parameters, since each parameter required a proper EMG signal of a different combination of muscles. The relatively small sample sizes at the ages of 4 and 10 months limits interpretation of the data. On the other hand, it should be realized that this is a frequently met limitation in this type of infant research (cf. Hadders-Algra et al. 1996a; Van der Fits et al. 1999b). Second, the results at the age of 18 months could have been influenced by the slightly different position of the infants at this age. In order to examine the possible size of this effect, we also explored data from the same infants sitting on the floor without support at 10 and 18 months (limited data, therefore not reported in detail). These data resembled those of the supported sitting condition reported earlier, for instance, direction specificity at trunk level still increased from 56 to 76 % (median values) between 10 and 18 months. Since this (larger) difference in sitting position did not influence the results to a great extent, it is unlikely that the minor change in sitting position introduced by lap sitting was the main cause of the different results at 18 months. Third, studying infants in a natural setting automatically implies limited control of the experimental set-up. As a result, variation in the initial position of the infant and in reaching behaviour may have influenced postural activity. Fourth, theoretically, the data could have been influenced by the change of recording software. However, since data analysis was performed on raw data in an identical fashion in both cases, we think this influence is negligible.

In this study, we chose to group the infants according to age, not their stage of development. Although improvement of postural strategies will undoubtedly influence sitting and reaching skills, we did not find significant relationships between sitting and/or reaching ability and our postural parameters. This may indicate that the development of postural control is a continuous process which at some point results in the achievement of a clinically observable milestone, that is, changes in discontinuous end states are not necessarily related to changes in the underlying continuous developmental process (Adolph and Robinson 2008).

Postural control between 4 and 18 months of age was characterized by variation, which was reflected in the variation in all parameters studied (direction specificity, the combinations in which the postural muscles were recruited, the latency of recruitment, the order in which they were recruited, and the use of anticipatory postural adjustments). This is in line with the idea that variation and variability are the hallmarks of typical development (Dusing and Harbourne 2010; Hadders-Algra 2010). The variation in all postural parameters fits to the framework of the Neuronal Group Selection Theory (NGST), where infants move from a phase of primary variability, during which they show variation and cannot adapt motor behaviour, to a phase of secondary variability during which they learn by experience to select optimal strategies from the varied repertoire, i.e. they develop variability (Hadders-Algra 2010). Remarkably, by the age of 4 months, we already found some early signs of variability with selection of the complete pattern and a slight preference for top-down recruitment. Our data are in line with the data of Hedberg et al. (2005) that the selection of the complete pattern starts after the age of 3 months. The higher frequency of the complete pattern at the age 4 months in our study compared to that reported by De Graaf-Peters et al. (2007) may be due to the improved performance of our onset detection algorithm.

The preference of top-down recruitment at the age of 4 months, the age of the emergence of reaching movements, may indicate the importance of stabilization of the head in space during this first active exploration of the environment (Hadders-Algra 2008). Indeed, Thelen and Spencer (1998) suggested that head stability plays an important role in the emergence of successful reaching. The possible association at the age of 6 months between top-down recruitment and the development of sitting and grasping also suggests that the head is an important frame of reference for postural control in early infancy. The gradual replacement by a bottom-up preference during the second half of infancy indicates that the focus of control moves towards the support surface, that is, in the lower trunk (Assaiante 1998; Hadders-Algra 2008).

The finding of an inconsistent use of direction-specific postural adjustments during reaching in infancy is in line with De Graaf-Peters et al. (2007), but at variance with the Van der Fits studies (Van der Fits and Hadders-Algra 1998; Van der Fits et al. 1999a, b). The presence of consistent direction specificity in the latter studies may be attributed to a more lenient definition of direction specificity. Direction-specific postural activity occurred more often in the trunk muscles than in the neck muscles. The lower rate of direction specificity in the neck may be attributed to the double function of the neck during reaching for a toy: infants do not only use neck muscles to stabilize their head in space, but they also may move their head towards the toy (Sveistrup et al. 2008). Interestingly, the increase in direction-specific trunk muscle activation between 10 and 18 months concurs with the increase in direction specificity observed during postural adjustments in response to external perturbation in a standing position (Hedberg et al. 2007). It could be surmised that the general increase in ability to recruit direction-specific activity is related to the development of independent stance and walking. However, even at 18 months, direction specificity is not a consistent finding during reaching whilst sitting supported. The fact that it is consistently present during reaching whilst sitting without support (Van der Heide et al. 2003) illustrates that the use of direction specificity is situation specific, that is, it depends on the degree to which the condition threatens balance control (Hadders-Algra 2008). Infants as young as 1 month consistently recruit direction-specific muscle activity during sitting when balance is vigorously perturbed (Hedberg et al. 2004, 2005), but our data indicate that the need to do so is considerably less in the rather safe situation of reaching during supported sitting.

Our finding of anticipatory activation in approximately one-third of the trials throughout the first year agrees with that of Van der Fits et al. (1999b). Our observation of the presence of some anticipatory postural activity in early infancy corresponds to reports on the ability of young infants to show anticipatory visuomotor behaviour (Von Hofsten et al. 1998, 2007). Unlike the studies of Van der Fits et al. (1999b), we did not find an increase in anticipatory activity at 15–18 months. Again, the difference in outcome between the studies most likely can be explained by methodological differences: Van der Fits et al. reported on anticipatory activity of dorsal and ventral postural muscles in a time window of 200 ms prior to the onset of the deltoid muscle, whereas we reported on activity of the direction-specific dorsal muscles in an anticipatory window of 100 ms before activation of the first arm muscle (deltoid, biceps, triceps, or pectoralis major).

## Concluding remarks

This study illustrated that postural activity in infants during the ecological task of reaching whilst sitting supported is characterized by a large variation. Yet within the variation two developmental trends could be distinguished: postural adjustments were more often direction-specific at 18 months than at younger ages. In addition, infants increasingly preferred bottom-up recruitment to top-down recruitment. More insight in postural strategies during reaching may be provided by studies combining an ecological study design, EMG measurements and kinematic and/or centre of pressure measurements.
